# Children and Adolescents in the United States with Usual High Added Sugars Intake: Characteristics, Eating Occasions, and Top Sources, 2015–2018

**DOI:** 10.3390/nu15020274

**Published:** 2023-01-05

**Authors:** Sohyun Park, Lixia Zhao, Seung Hee Lee, Heather C. Hamner, Latetia V. Moore, Deborah A. Galuska, Heidi M. Blanck

**Affiliations:** Division of Nutrition, Physical Activity, and Obesity (DNPAO), National Center for Chronic Disease Prevention and Health Promotion (NCCDPHP), Centers for Disease Control and Prevention (CDC), Atlanta, GA 30341, USA

**Keywords:** added sugars, sugar-sweetened beverages, US children and adolescents, youth, diet

## Abstract

Background: High consumption of added sugars is related to adverse health consequences. Objective: The objective of this study was to examine characteristics of US youth who report high intakes of added sugars, as well as the eating occasions and top sources of added sugars that contributed to intakes among consumers with high added sugars intake. Design and participants/setting: We conducted a cross-sectional study using 2015–2018 NHANES data among 5280 US youths (2–19 years). Main outcome measures: Outcome measure was usual percent of calories from added sugars using 2 days of dietary recall based on the National Cancer Institute method. High consumers were defined as consuming greater than 15% of total daily calorie intake from added sugars (1.5 times higher than the 2020–2025 Dietary Guidelines for Americans recommendation of <10% of total daily calorie intake). Explanatory measures were selected sociodemographics (e.g., age, sex, race/ethnicity). Eating occasions were breakfast, lunch, dinner, and snack. Statistical analyses performed: We used *t*-tests to compare mean differences between sociodemographic groups. Results: Overall, 34% of US youths were classified as high consumers of added sugars. The prevalence of high consumers of added sugars significantly varied by some sociodemographics (i.e., age, race/ethnicity, and head of household’s education level). The prevalence of high added sugars consumers was significantly greater among 12–19-year-olds (41%) and 6–11-year-olds (37%) compared to 2–5-year-olds (19%), non-Hispanic Black (42%) and non-Hispanic White (42%) persons compared to Hispanic persons (19%), and those with a head of household’s education level of high school/some college (40%) compared to households with college degree or higher (29%). The prevalence of high consumers did not differ by sex, income, or weight status. Of eating occasions, the amount of added sugars youths consumed was highest during snack occasions among high consumers. Top five sources of added sugars among high consumers on a given day were sweetened beverages, sweet bakery products, candy, other desserts, and ready-to-eat cereals. Conclusion: One in three US youths consumed more than 15% of total calories from added sugars. High added sugars intake was more prevalent among certain subgroups such as 12–19-year-olds and non-Hispanic Black or non-Hispanic White youth. Our findings can provide information for intervention efforts to decrease added sugars intake to promote child health.

## 1. Introduction

High prevalence of obesity among US children and adolescents [1] is a public health issue. In 2017–March 2020, 22% of US youths (2–19 years) had obesity, and obesity prevalence increases with age [1]. In 2017–March 2020, 13% of US children aged 2–5 years, 23% of children aged 6–11 years, and 26% of adolescents aged 12–19 had obesity [1]. Greater intake of added sugars, including sugar-sweetened beverages (SSB), is linked to obesity and other health consequences including dyslipidemia and cardiovascular disease risk in youth [2,3,4,5]. Added sugars are defined as sugars that are inserted through the processing of foods and include sweeteners (e.g., table sugar), syrups, honey, and sugars from concentrated fruit or vegetable juice [6]. The 2020–2025 Dietary Guidelines for Americans (DGA) recommend that Americans aged ≥2 years should consume less than 10% of total daily calories from added sugars, and children aged <2 years should avoid consuming added sugars [7]. Although the consumption of added sugars among US children and adolescents has decreased over time [8], intake remains high. Overall, US youth (2–19 years) consumed on average 14% of total daily calories from added sugars in 2015–2016, and only 35% of US youths (2–19 years) met the DGA recommendation for limiting added sugars [9]. The top three sources of added sugars among US youths (2–19 years) were sweetened beverages, sweet bakery products, and candy in 2015–2016 [9]. Based on the 2017–2018 National Health and Nutrition Examination Survey (NHANES), mean intakes of added sugars were 12 teaspoons (tsp)/day (50 g) for children aged 2–5 years, 17 tsp/day (71 g) for children aged 6–11 years, and 18 tsp/day (75 g) for adolescents aged 12–19 years [10].

To address concerns of high consumption of added sugars among Americans, one of the US Department of Health and Human Services’ Healthy People 2030 Objectives is “Reduce consumption of added sugars by people aged 2 years and over” [11]. Although several studies have examined consumption patterns and top sources of added sugars among all US youths [7,8,9,12], there is limited information on characteristics, leading sources, and eating occasions of added sugars among high consumers of added sugars. As higher intake of added sugars is related to adverse health consequences in youth [2,3,4,5], targeting interventions for the high consumer group might have a greater impact on health. Thus, the objective of this study was to examine characteristics of US children and adolescents (2–19 years) who report high intakes of added sugars, as well as the eating occasions and top sources of added sugars that contributed to intakes among consumers with high added sugars intake.

## 2. Materials and Methods

### 2.1. Data Source and Participants

NHANES is program of the National Center for Health Statistics (NCHS), Centers for Disease Control and Prevention (CDC). This study used data from the NHANES 2015–2018. NHANES is a cross-sectional, nationally representative, complex survey with a multistage cluster sampling design of the US civilian, noninstitutionalized population [13]. The survey combines home interviews, an in-person physical examination and a 24 h dietary recall conducted in a mobile examination center, and a second 24 h dietary recall collected by telephone 3–10 days later. For children aged 2–5 years, dietary intake was reported by a proxy, and for children aged 6–11 years, dietary intake was reported by participants with assistance of a proxy. Dietary intake was self-reported by participants aged ≥12 years. NHANES is approved by NCHS Ethics Review Board. Publicly available datasets exclude personal identifiers; thus, the current study was exempt from the CDC institutional review board.

### 2.2. Outcome Variables

US Department of Agriculture (USDA)’s Food Patterns Equivalents Database (FPED) for 2015–2016 and 2017–2018 [10,14] were used to disaggregate all foods and beverages into their components and quantify added sugars intake. We merged individual food files from NHANES 2015–2016 and 2017–2018 with the corresponding FPED to obtain information on added sugars for each food. The total daily amount of added sugars intake for each participant, in tsp equivalents, were summed across all foods and beverages that were reported. We converted tsp equivalents into grams by multiplying by 4.2 g/tsp equivalent, then calculated calories from added sugars by multiplying 4 kcal/grams. To obtain the percentage of calories (% kcal) from added sugars, we divided the calories from added sugars by the total energy intake (in kcal/day), which was provided in NHANES dietary data files. The calculations were done separately for the first and second 24 h dietary recalls. We defined high added sugars consumers as those who consumed greater than 15% of total calories from added sugars, which is 1.5 times higher than the DGA recommendation (i.e., <10% of total daily calorie intake). 

We used the National Cancer Institute (NCI) [15] method to estimate usual mean total energy intake and proportion of participants who consumed greater than 15% of total calories from added sugars (hereafter, referred to as high consumers). Because nearly every participant consumed added sugars and energy daily, we employed the linear mixed-effects models to estimate the amount of the food or nutrient consumed accounting for weekend vs. weekday effects. The NCI method requires that all participants have at least 1 day of dietary recall and some of them have multiple days of dietary recalls to estimate the between- and within-person variation in intake [15]. The analytic sample size was determined by the total number of respondents with a Day 1 dietary recall when using NHANES data. Of the 5281 participants aged 2–19 years with a completed reliable in-person 24 h (Day 1) dietary recall, one participant with missing added sugars intake on the Day 1 dietary recall (*n* = 1) was excluded, leaving a sample of 5280 children and adolescents for the current analysis. In our study, 4362 (81.9%) participants provided two reliable 24 h dietary recalls.

### 2.3. Explanatory Variables

Explanatory variables included age (2–5 years, 6–11 years, 12–19 years), sex (boys, girls), race/ethnicity (non-Hispanic [NH] Black, Hispanic, NH Other, NH White), head of household education (less than high school, high school graduate/GED or some college, college graduate or above), federal poverty income ratio (<130%, 130% to 350%, ≥350%) [16]. Federal poverty income ratio is the ratio of family income to the US Department of Health and Human Services’ poverty threshold for the specific survey year, which was calculated by dividing family income by the poverty guidelines specific to the survey year [17]. Measured weight and height were used to calculate body mass index (BMI) which is calculated by dividing a person’s weight in kilograms by the square of height in meters. Weight status was categorized as underweight/healthy weight (BMI < 85th percentile), overweight (BMI 85th to <95th percentile), or obesity (BMI ≥ 95th percentile), in relation to the 2000 Centers for Disease Control and Prevention growth charts age- and sex-specific percentiles [18]. Because only about 3% of children and adolescents were classified as underweight, we combined underweight with healthy weight into one category.

We used the Day 1 24 h dietary recall to identify high consumers (*n* = 2144) and to examine eating occasions related to added sugars and sources of added sugars among high consumers, because it is not recommended to use estimated usual intake to classify individuals into categories [19]. For each food and beverage reported, a list of 20 different names of eating occasions were provided to the participant for selection. We categorized those eating occasions as four categories: (1) breakfast (e.g., breakfast, desayuno, and almuerzo); (2) lunch (e.g., lunch, comida, and brunch); (3) dinner (e.g., dinner, supper, and cena); (4) snack (e.g., snack, merienda, entre comida, botana, bocadillo, tentempie, extended consumption, drink, and bebida). For each eating occasion, the amounts of added sugars (in kcal) intake for each participant were summed across all foods and beverages reported at the eating occasion. The percentage (%) contribution of added sugars for each eating occasion was calculated as the sum of the amount of added sugars consumed at the eating occasion for all participants, divided by the sum of added sugars consumed from all foods and beverages for all participants multiplied by 100.

To identify top food sources/groups contributing to added sugars consumption, USDA What We Eat in America food categories for each NHANES cycle were collapsed into 18 food groups: Bread/rolls/tortillas, candy, condiments and sauces, cooked grains and cereals, cured meats/poultry, fats and oils, flavored milk/milk substitutes, jams/syrups/sugars, mixed dishes, other desserts, protein foods, quick breads and bread products, ready-to-eat cereals, snack/meal bars and crackers, sweet bakery products, sweetened beverages, yogurt, and other. Food groups were ranked based on their percentage contribution to total added sugars intake on Day 1 dietary data, calculated as the sum of added sugars consumed from a specific food group divided by the sum of added sugars consumed from all food groups and multiplied by 100.

### 2.4. Statistical Analysis

Descriptive analyses were presented as weighted means and standard errors (SE) or weighted percentage for sociodemographic characteristics and weight status. T-tests were used to compare mean differences of usual total energy intake or % calories from added sugars between groups (e.g., boys vs. girls, aged 2–5 years vs. 6–11 years, NH White vs. Hispanic persons). All tests were two-sided, and *p*-values < 0.05 were considered statistically significant. All statistical analyses were performed in SAS 9.4 (SAS Institute Inc, Cary, NC, USA) or SAS-callable SUDAAN (RTI International, Research Triangle Park, NC, USA) using combined Day 1 dietary sample weights from two survey cycles (2015–2016 and 2017–2018) to account for non-response and the complex sampling design. 

## 3. Results

Overall, in 2015–2018, about half of US youths were 12- to 19-year-olds (45%), boys (51%), and NH White persons (51%). About 28% had a head of household education of college or higher, 29% had federal poverty income ratio ≥350%, and 20% had obesity (Table 1). In 2015–2018, US youths aged 2–19 years consumed an average of 1828 kcal per day, and usual mean total energy intake differed significantly by age, sex, race/ethnicity, and federal poverty income ratio (*t*-tests, *p* < 0.05; Table 1). In 2015–2018, 34% of US children and adolescents were classified as high consumers (Table 1). The prevalence of high consumers differed significantly by certain sociodemographic characteristics (i.e., age, race/ethnicity, and head of household education) (*t*-tests, *p* < 0.05). However, the prevalence of high consumers did not differ by sex, federal poverty income ratio, or weight status (Table 1).

By eating occasions among high consumers, mean calories of added sugars intake were highest during snack occasions (177 kcal/day), followed by dinner time (98 kcal/day), lunch time (86 kcal/day) and breakfast time (72 kcal/day) on a given day (Table 2). Overall, 41% of added sugars were consumed during snack occasions, followed by dinner time (23% contribution of added sugars), lunch time (20% contribution of added sugars), and breakfast time (17% contribution of added sugars). Similar patterns were observed by age groups except 2–5-year-olds (Table 2). The lowest percentage of contribution of added sugars was lunch time for 2–5-year-olds, while it was breakfast time for other age groups (Table 2).

The top five leading sources of added sugars (together providing about 80% of added sugars) among high consumers on a given day were sweetened beverages (186 kcal/day, 43% of added sugars calories), sweet bakery products (66 kcal/day, 15% of added sugars calories), candy (40 kcal/day, 9% of added sugars calories), other desserts (31 kcal/day, 7% of added sugars calories), and ready-to-eat cereals (22 kcal/day, 5% of added sugars calories) (Table 3). The same patterns were observed among all age groups except 2–5-year-olds. While the top three sources of added sugars were the same for all age groups, the 4th and 5th top sources of added sugars were flavored milk/milk substitutes and other desserts for high consumers aged 2–5 years, whereas they were other desserts and ready-to-eat cereals for other age groups (Table 3). 

## 4. Discussion

In 2015–2018, one in three US youths aged 2–19 years old consumed more than 15% of total daily calories from added sugars. Additionally, the prevalence of high consumers significantly differed by age, race/ethnicity, and head of household education among US children and adolescents. For instance, the prevalence of high consumers was significantly higher among adolescents (vs. younger children), NH Black or NH White children (vs. Hispanic children), and children with a head of household education of high school or some college (vs. college or higher) in 2015–2018. Similar to our findings, a previous study reported that 6–11-year-olds (31%) and 12–19-year-olds (31%) were less likely to meet 2020–2025 DGA added sugars recommendation to limit added sugars to less than 10% of calories than 2–5-year-olds (47%) according to 2015–2016 NHANES data [9].

Consistent with our results, a previous study using 2003–2010 NHANES data reported that NH White children and adolescents (24 tsp/day [100 g]) had significantly higher added sugars intake than Mexican-American children and adolescents (20 tsp/day [83 g]) aged 6–19 years [12]. Based on 2017–2018 NHANES data, mean added sugars intakes were lower among Hispanic children and adolescents (16 tsp/day [67 g] for 6–11-year-olds; 15 tsp/day [63 g] for 12–19-year-olds) compared with NH White children and adolescents (18 tsp/day [75 g] for 6–11-year-olds; 20 tsp/day [83 g] for 12–19-year-olds) and NH Black children and adolescents (19 tsp/day [79 g] for 6–11-year-olds; 20 tsp/day [83 g] for 12–19-year-olds) [10], which were also consistent with our findings. However, inconsistent with our findings, a previous study found that added sugars intake was significantly higher among NH White children and adolescents (24 tsp/day [100 g]) than NH Black children and adolescents (22 tsp/day [92 g]) aged 6–19 years in 2003–2010 [12]. In our study, the prevalence among US children and adolescents of high consumers did not differ by sex, family income, and weight status. Similar to our findings, previous studies also reported that added sugars intake did not differ by family income status [10,12]. However, a previous report showed that boys had higher mean added sugars intake (18 tsp/day [75 g]) than girls aged 2–19 years (15 tsp/day [63 g]) in 2017–2018 [10]. Discrepancies in study findings could be partially due to a methodology difference in that we calculated usual intake using 2 days of dietary recalls whereas other studies used 1-day dietary recall. Another difference could be related to focusing on high consumers in our study.

By eating occasions, we found that mean calorie intake of added sugars was highest during snack occasions among high consumers, which contributed 41% of added sugars calories in 2015–2018. For adolescents (12–19 years), added sugars intake was also high during dinner in addition to snack occasions in our study. Based on 2009–2016 NHANES data, US adolescents (12–19 years) consumed 2.4 snacks/day on a given day, and most snacks were consumed at home (consuming 1.7 snacks/day at home and 0.7 snacks/day away from home) [20]. Another study reported that the majority of added sugars consumed by school aged children came from grocery stores, followed by restaurants and school cafeteria in 2003–2010 [12]. Because snacks were commonly consumed by US children [20] and most added sugars were consumed during snack occasions, intervention strategies may focus on low- or no- added sugars snacks in efforts to reduced added sugars intake, which might have even more impact on children who are high consumers. Schools have made good progress on changing the school environment to support the reduction of added sugars intake among US children intake [21]; such as by limiting purchases of regular soda or fruit drinks during the school day or on school campuses [22]. Continuing these efforts with a focus on providing healthy snacks (e.g., fruits and vegetables) during snack occasions could further reduce added sugars intake among all groups of high consumers.

In our study, the top five leading sources of added sugars among high consumers on a given day were sweetened beverages, sweet bakery products, candy, other desserts, and ready-to-eat cereals. For instance, we found that almost half (43%) of added sugars calories came from sweetened beverages among high consumers aged 2–19 years on a given day in 2015–2018. A previous study reported that the top three leading sources of added sugars among all US youths (2–19 years) were SSB, sweet bakery products, and candy in 2015–2016 [9]. Similarly, we found that the top three sources of added sugars were the same for all high consumers: sweetened beverages, sweet bakery products, and candy. Although the percentage of calorie intake from SSB decreased from 1999–2000 to 2017–2018, the percentage of calorie intake from sweet snacks/sweets increased during the same time period among US youth (2–19 years) [23]. Moreover, characteristics of children and adolescents who consumed SSB are different from those who consume sweet foods. For instance, SSB intake was higher among adolescents (12–19 years vs. younger children), boys (vs. girls), and those who live in the lowest family income household (vs. highest income household) based on NHANES data from 2003 to 2018 [16,24,25,26,27]. However, sweet food intake (e.g., sweet bakery products, candy, and other desserts) was higher in younger children (vs. adolescents) and those who live in the highest family income group (vs. lowest income group), and sweet food intake was not different by sex among US children [28]. 

One strength of our study is use of the NCI methods on calculating usual intake to identify high consumers of added sugars. By using two days of 24 h dietary recall data to identify high consumers, this analysis reflected usual added sugars intake among US children and adolescents after considering individual variances. Another is the use of large, nationally representative samples of US children and adolescents. This study has four limitations. First, we used one 24 h dietary recall data to identify eating occasions and top sources of added sugars among high consumers. Thus, eating occasions and top sources may not represent high consumers’ usual consumption patterns. Second, findings might be subject to potential reporting bias and/or recall bias. Third, dietary intake data for younger children may be underestimated or overestimated because caregivers may not observe all eating occasions. Fourth, NHANES is cross-sectional, and therefore causality cannot be inferred. 

## 5. Conclusions

Results suggest that one in three US children and adolescents were classified as high consumers of added sugars in 2015–2018. The prevalence of high consumers among US children and adolescents varied by age, race/ethnicity, and head of household education. Among high consumers, most of added sugars were consumed during snack occasions, and the top five sources of added sugars on a given day were sweetened beverages, sweet bakery products, candy, other desserts, and ready-to-eat cereals. Our findings can guide the development of interventions to reduce high added sugars consumption among children and adolescents and thus support their health.

## Figures and Tables

**Table 1:** Characteristics of the survey population and their daily mean total energy intake and participants consuming over 15% energy from added sugars (i.e., high consumers) among US children and adolescents aged 2–19 years, NHANES 2015–2018, based on usual intake method.

Characteristics		Prevalence		Usual Mean Total Energy Intake		Consuming >15% Energy from Added Sugars	
	*n* ^a^	% ^b^	SE	kcal/Day	95% CI	Prevalence %	95% CI
**Total**	5280	100		1828	1805, 1852	34.4	31.0, 37.9
**Age**							
2–5 years (Reference)	1205	21.4	0.7	1488	1460, 1515	18.6	14.3, 22.8
6–11 years	1835	33.4	0.9	1873 *	1841, 1904	37.2 *	31.7, 42.6
12–19 years	2240	45.1	1.4	1960 *	1911, 2009	40.9 *	36.1, 45.7
**Sex**							
Boys (Reference)	2636	50.9	1.1	1968	1933, 2004	34.5	30.6, 38.5
Girls	2644	49.1	1.1	1685 *	1660, 1711	34.7	30.3, 39.1
**Race/ethnicity**							
Black, non-Hispanic	1185	13.3	1.9	1828	1771, 1885	41.8 *	36.4, 47.2
Hispanic (Reference)	1565	24.7	2.7	1759	1715, 1803	18.5	12.1, 24.8
Other, non-Hispanic	902	11.3	1.0	1850 *	1804, 1897	26.2	21.1, 31.3
White, non-Hispanic	1628	50.7	3.4	1863 *	1824, 1902	41.6 *	36.4, 46.9
**Head of household education**							
<High school	1081	17.6	1.9	1782	1710, 1853	30.0	21.8, 38.2
High school or Some college	2834	54.3	1.7	1824	1783, 1864	39.6 *	35.6, 43.7
College or higher (Reference)	1123	28.2	2.2	1879	1825, 1933	29.0	23.6, 34.3
**Federal poverty income ratio ^c^**							
<130%	1937	32.0	1.9	1780 *	1729, 1830	34.2	30.2, 38.1
130% to <350%	1890	39.0	1.6	1841	1796, 1887	37.5	33.5, 41.4
≥350% (Reference)	992	29.0	2.2	1888	1829, 1946	31.7	24.8, 38.6
**Weight status ^d^**							
Underweight /healthy weight (Reference)	3253	63.6	1.2	1843	1813, 1874	34.4	30.2, 38.6
Overweight	848	16.1	0.7	1795	1740, 1850	34.2	29.1, 39.4
Obesity	1095	20.3	1.0	1838	1781, 1894	35.8	29.0, 42.5

Abbreviations: SE, standard error; CI, confidence interval; NHANES, National Health and Nutrition Examination Survey. * Significantly different from the reference group (*p* < 0.05). ^a^ Unweighted sample size. ^b^ Weighted percent may not add up to 100% because of rounding. ^c^ Federal poverty income ratio, is the ratio of family income to poverty, which was calculated by dividing family income by the poverty guidelines specific to the survey year. ^d^ Weight status was based on calculated body mass index (BMI) (kg/m^2^) from measured weight and height data: underweight/healthy weight (BMI < 85th percentile), overweight (BMI 85th to 95th percentile), or obesity (BMI ≥ 95th percentile), in relation to the 2000 Centers for Disease Control and Prevention growth charts age-and sex-specific percentiles.

**Table 2:** Added sugars consumption by age and eating occasions among high consumers aged 2–19 years, NHANES 2015–2018, on a given day (based on Day 1 dietary recall (*n* = 2144)) ^a^.

Eating Occasion	Among High Added Sugars Consumers ^b^	
	Mean Added Sugars (kcal/Day) ± SE	% of Contribution ^c^ ± SE
**All children and adolescents**		
Breakfast	71.8 ± 3.0	16.6 ± 0.6
Lunch	85.9 ± 2.9	19.8 ± 0.7
Dinner	97.7 ± 4.1	22.6 ± 0.9
Snack	177.3 ± 5.7	41.0 ± 1.1
**2–5 years**		
Breakfast	62.6 ± 6.0	19.2 ± 1.5
Lunch	50.6 ± 4.4	15.5 ± 1.4
Dinner	63.2 ± 6.7	19.4 ± 2.1
Snack	149.9 ± 10.8	45.9 ± 2.5
**6–11 years**		
Breakfast	69.4 ± 3.5	16.4 ± 0.8
Lunch	88.2 ± 4.2	20.9 ± 1.0
Dinner	84.8 ± 6.0	20.1 ± 1.3
Snack	180.3 ± 7.6	42.7 ± 1.6
**12–19 years**		
Breakfast	76.5 ± 5.0	16.2 ± 1.0
Lunch	95.3 ± 4.0	20.1 ± 0.9
Dinner	118.2 ± 5.4	24.9 ± 1.1
Snack	183.7 ± 9.3	38.8 ± 1.5

Abbreviations: SE, standard error; NHANES, National Health and Nutrition Examination Survey. ^a^ The Day 1 24 h dietary recall was used to identify high consumers (*n* = 2144). ^b^ High consumers were defined as those who consumed greater than 15% of total calories from added sugars, which is 1.5 times higher than Dietary Guidelines for Americans recommendation (i.e., <10% of total daily calorie intake). ^c^ The percentage (%) of added sugars consumed was calculated as the sum of the amount of added sugars from the foods and beverages consumed at each eating occasion for all persons in the designated group, divided by the sum of added sugars consumed from all foods and beverages for all persons in the designated group multiplied by 100.

**Table 3:** Top five sources of added sugars and mean calorie intake from top five sources among high consumers of added sugars ^a^ aged 2–19 years, overall and by age, NHANES 2015–2018, on a given day (based on Day 1 dietary recall (*n* = 2144)) ^b^.

Ranking	Food Category	Mean Added Sugars (kcal/Day) ± SE	% ± SE of Added Sugars ^c^
**All high consumers aged 2–19 years**			
1	Sweetened Beverages	186.3 ± 5.8	43.1 ± 1.1
2	Sweet Bakery Products	65.6 ± 3.5	15.2 ± 0.7
3	Candy	40.4 ± 3.2	9.3 ± 0.7
4	Other Desserts	31.2 ± 2.6	7.2 ± 0.6
5	Ready-to-eat cereals	22.5 ± 1.2	5.2 ± 0.3
**High consumers aged 2–5 years**			
1	Sweetened Beverages	106.1 ± 9.6	32.5 ± 2.4
2	Sweet Bakery Products	61.2 ± 6.8	18.7 ± 1.7
3	Candy	33.5 ± 3.7	10.3 ± 1.1
4	Flavored milk/Milk substitutes	30.8 ± 4.9	9.4 ± 1.4
5	Other Desserts	26.4 ± 4.8	8.1 ± 1.6
**High consumers aged 6–11 years**			
1	Sweetened Beverages	148.5 ± 6.5	35.1 ± 1.4
2	Sweet Bakery Products	72.3 ± 3.8	17.1 ± 0.8
3	Candy	49.1 ± 5.8	11.6 ± 1.3
4	Other Desserts	36.7 ± 3.4	8.7 ± 0.8
5	Ready-to-eat cereals	24.5 ± 1.6	5.8 ± 0.4
**High consumers aged 12–19 years**			
1	Sweetened Beverages	239.8 ± 7.4	50.6 ± 1.5
2	Sweet Bakery Products	62.1 ± 5.9	13.1 ± 1.2
3	Candy	36.2 ± 4.3	7.6 ± 0.9
4	Other Desserts	28.7 ± 3.5	6.1 ± 0.7
5	Ready-to-eat cereals	23.1 ± 2.4	4.9 ± 0.5

Abbreviations: SE, standard error; NHANES, National Health and Nutrition Examination Survey. ^a^ High consumers were defined as those who consumed greater than 15% of total calories from added sugars, which is 1.5 times higher than Dietary Guidelines for Americans recommendation (i.e., <10% of total daily calorie intake). ^b^ The Day 1 24 h dietary recall was used to identify high consumers (*n* = 2144). ^c^ The population proportion (%) of added sugars is defined as the sum of the added sugars consumed from each specific food category for all participants in the designated group, divided by the sum of added sugars consumed from all the food categories for all participants in the same group, multiplied by 100.

## Data Availability

NHANES data are publicly available in the NCHS, CDC (https://wwwn.cdc.gov/nchs/nhanes/Default.aspx).

## References

[1] Hu K., Staiano A.E. (2022). Trends in Obesity Prevalence among Children and Adolescents Aged 2 to 19 Years in the US from 2011 to 2020. JAMA Pediatr..

[2] Vos M.B., Kaar J.L., Welsh J.A., Van Horn L.V., Feig D.I., Anderson C.A.M., Patel M.J., Cruz Munos J., Krebs N.F., Xanthakos S.A. (2017). Added Sugars and Cardiovascular Disease Risk in Children: A Scientific Statement From the American Heart Association. Circulation.

[3] Welsh J.A., Sharma A., Cunningham S.A., Vos M.B. (2011). Consumption of added sugars and indicators of cardiovascular disease risk among US adolescents. Circulation.

[4] DiNicolantonio J.J., Lucan S.C. (2014). The wrong white crystals: Not salt but sugar as aetiological in hypertension and cardiometabolic disease. Open Heart.

[5] Zhang Z., Gillespie C., Welsh J.A., Hu F.B., Yang Q. (2015). Usual intake of added sugars and lipid profiles among the U.S. adolescents: National Health and Nutrition Examination Survey, 2005–2010. J. Adolesc. Health.

[6] Dietary Guidelines Advisory Committee (2020). Scientific Report of the 2020 Dietary Guidelines Advisory Committee: Advisory Report to the Secretary of Agriculture and the Secretary of Health and Human Services.

[7] U.S. Department of Agriculture, U.S. Department of Health and Human Services Dietary Guidelines for Americans, 2020–2025. 9th ed. December 2020. https://www.dietaryguidelines.gov/sites/default/files/2020-12/Dietary_Guidelines_for_Americans_2020-2025.pdf.

[8] Bowman S.A., Clemens J.C., Friday J.E., Schroeder N., Shimizu M., LaComb R.P., Moshfegh A.J. (2018). Food Patterns Equivalents Intakes by Americans: What We Eat in America, NHANES 2003–2004 and 2015–2016. Food Surveys Research Group. Dietary Data Brief No. 20. https://www.ars.usda.gov/ARSUserFiles/80400530/pdf/DBrief/20_Food_Patterns_Equivalents_0304_1516.pdf.

[9] Bowman S.A., Clemens J.C., Friday J.E., Schroeder N., LaComb R.P. (2019). Added Sugars in American Children’s Diet: What We Eat in America, NHANES 2015–2016. Food Surveys Research Group. Dietary Data Brief No. 26. https://www.ars.usda.gov/ARSUserFiles/80400530/pdf/DBrief/26_Sources%20of%20Added%20Sugars%20in%20Children's%20Diet_1516.pdf.

[10] U.S. Department of Agriculture Food and Nutrition Service, Agricultural Research Service. 2020. Food Patterns Equivalents Intakes from Food: Mean Amounts Consumed per Individual, What We Eat in America, NHANES 2017–2018. https://www.ars.usda.gov/ARSUserFiles/80400530/pdf/FPED/Tables_1-4_FPED_1718.pdf.

[11] U.S. Department of Health and Human Services Healthy People 2030. Overweight and Obesity. https://health.gov/healthypeople/objectives-and-data/browse-objectives/overweight-and-obesity.

[12] Drewnowski A., Rehm C.D. (2014). Consumption of added sugars among US children and adults by food purchase location and food source. Am. J. Clin. Nutr..

[13] U.S. Centers for Disease Control and Prevention About the National Health and Nutrition Examination Survey. https://www.cdc.gov/nchs/nhanes/about_nhanes.htm.

[14] U.S. Department of Agriculture (2018). Agricultural Research Service. Food Patterns Equivalents Intakes from Food: Mean Amounts Consumed per Individual, by Gender and Age, What We Eat in America, NHANES 2015–2016. https://www.ars.usda.gov/ARSUserFiles/80400530/pdf/FPED/tables_1-4_FPED_1516.pdf.

[15] Tooze J.A., Kipnis V., Buckman D.W., Carroll R.J., Freedman L.S., Guenther P.M., Krebs-Smith S.M., Subar A.F., Dodd K.W. (2010). A mixed-effects model approach for estimating the distribution of usual intake of nutrients: The NCI method. Stat. Med..

[16] Gangrade N., Figueroa J., Leak T.M. (2021). Socioeconomic disparities in foods/beverages and nutrients consumed by U.S. adolescents when snacking: National Health and Nutrition Examination Survey 2005–2018. Nutrients.

[17] U.S. Centers for Disease Control and Prevention National Health and Nutrition Examination Survey 2017–2018 Data Documentation, Codebook, and Frequencies. https://wwwn.cdc.gov/Nchs/Nhanes/2017-2018/DEMO_J.htm.

[18] Kuczmarski R.J., Ogden C.L., Grummer-Strawn L.M., Flegal K.M., Guo S.S., Wei R., Mei Z., Curtin L.R., Roche A.F., Johnson C.L. (2000). CDC growth charts: United States. Adv. Data.

[19] National Cancer Institute, Division of Cancer Control & Population Sciences Usual Dietary Intakes: Details of the Method. https://epi.grants.cancer.gov/diet/usualintakes/details.html.

[20] Casey C., Huang Q., Talegawkar S.A., Sylvetsky A.C., Sacheck J.M., DiPietro L., Lora K.R. (2021). Added sugars, saturated fat, and sodium intake from snacks among U.S. adolescents by eating location. Prev. Med. Rep..

[21] Poti J.M., Slining M.M., Popkin B.M. (2013). Solid fat and added sugar intake among U.S. children: The role of stores, schools, and fast food, 1994–2010. Am. J. Prev. Med..

[22] U.S. Centers for Disease Control and Prevention School Health Profiles 2018: Characteristics of Health Programs among Secondary Schools. https://www.cdc.gov/healthyyouth/data/profiles/pdf/2018/CDC-Profiles-2018.pdf.

[23] Wang L., Martínez Steele E., Du M., Pomeranz J.L., O’Connor L.E., Herrick K.A., Luo H., Zhang X., Mozaffarian D., Zhang F.F. (2021). Trends in consumption of ultraprocessed foods among US youths aged 2–19 years, 1999–2018. JAMA.

[24] Rosinger A.Y., Bethancourt H., Francis L.A. (2019). Association of caloric intake from sugar-sweetened beverages with water intake among US children and young adults in the 2011-2016 National Health and Nutrition Examination Survey. JAMA Pediatr..

[25] Bleich S.N., Vercammen K.A., Koma J.W., Li Z. (2018). Trends in beverage consumption among children and adults, 2003–2014. Obesity.

[26] Rosinger A., Herrick K., Gahche J., Park S. (2017). Sugar-sweetened Beverage Consumption among U.S. Youth, 2011–2014. NCHS Data Brief.

[27] Moshfegh A.J., Garceau A.O., Parker E.A., Clemens J.C. (2019). Beverage Choices among Children: What We Eat in America, NHANES 2015–2016. Food Surveys Research Group Data Brief No. 22. https://www.ars.usda.gov/ARSUserFiles/80400530/pdf/DBrief/22_Beverage_choices_children_1516.pdf.

[28] Martin C.L., Sebastian R.S., Enns C.W., Goldman J.D., Moshfegh A.J. Sweet Foods Consumption by Children in the U.S. What We Eat in America, NHANES 2015–2018. Food Surveys Research Group Dietary Data Brief No. 34. 2020. https://www.ars.usda.gov/ARSUserFiles/80400530/pdf/DBrief/34_Sweet_foods_children_1518.pdf.

